# Modified Implant Fixation Technique Is an Alternative for Patients with an Anterior Cruciate Ligament Tear in Limited Resource Settings: A Comparison Functional Outcome Study with Polyether Ether Ketone and Bioabsorbable Screws

**DOI:** 10.3390/jcm13102964

**Published:** 2024-05-17

**Authors:** Muhammad Sakti, Arian Fardin Ignatius Wawolumaja, Ruksal Saleh, Muhammad Andry Usman, Jainal Arifin, Muhammad Phetrus Johan

**Affiliations:** 1 Department of Orthopedic & Traumatology, Faculty of Medicine, Hasanuddin University, Makassar 90245, Indonesia; arian.wawolumaja@gmail.com (A.F.I.W.); ruksal_saleh@yahoo.com (R.S.); andryusman@med.unhas.ac.id (M.A.U.); jainalarifin.ot@gmail.com (J.A.); muhpjo@gmail.com (M.P.J.); 2 Department of Orthopedic & Traumatology, Wahidin Sudirohusodo Hospital, Makassar 90245, Indonesia

**Keywords:** anterior cruciate ligament, anterior cruciate ligament reconstruction, PEEK screws, Lysholm Knee Score, IKDC score

## Abstract

**Background:** Anterior cruciate ligament (ACL) injury is one of the most prevalent factors contributing to knee instability worldwide. This study aimed to evaluate modified metal fixation techniques for ACL reconstruction compared to factory-made implants, such as polyether ether ketone (PEEK) screws, bioabsorbable screws, and modified metal implants. **Methods:** A retrospective cohort analysis was conducted to assess the functional outcomes of ACL using various fixation methods. Patients who underwent arthroscopic ACL reconstruction at several healthcare facilities were included in the study. The functional outcomes were evaluated using the Lysholm Knee Scoring Scale and the International Knee Documentation Committee (IKDC) score questionnaire at 6- and 12 months post-surgery. Statistical analyses, including the Shapiro–Wilk test and analysis of variance, were performed to compare outcomes among the fixation groups. **Results:** Thirty-three patients who underwent ACL reconstruction surgery with varying distributions across the three fixation groups (modified metal implants, PEEK screws, and bioabsorbable screws) were included in the study. As measured by the Lysholm and IKDC scores at 6- and 12 months post-surgery, the PEEK group demonstrated the highest average scores. Nevertheless, these functional outcomes were not significantly different between the groups (*p* = 0.140, 0.770, 0.150, and 0.200). These findings align with those of meta-analyses comparing different fixation methods for ACL reconstruction. **Conclusions:** While acknowledging the small sample size as a limitation, this study suggests that modified metal implants represent viable options for ACL reconstruction. The selection of fixation methods should consider patient characteristics and preferences, emphasizing biomechanical stability and long-term outcomes. Further research is needed to validate these findings and explore their biomechanical properties and cost-effectiveness.

## 1. Introduction

Joint instability, particularly that affecting the knee joint, poses significant challenges for athletes and non-athletes. Anterior cruciate ligament (ACL) injury is one of the most prevalent factors contributing to knee instability worldwide. ACL injuries occur in the knee, with 129,000–200,000 reconstructions yearly in the United States and 400,000 worldwide [1,2].

ACL reconstruction surgery is the gold standard for ACL tear treatment. Multiple reconstruction techniques can be used to restore rotational and translational knee stability and function. Technical challenges during ACL reconstruction affect patients’ clinical outcomes, including graft selection, tunnel positioning, graft tensioning, fixation methods, and healing properties. Several devices have been developed for graft fixation, in addition to a wide array of fixation methods. The different types of fixation methods used in ACL reconstruction are typically categorized as compression, suspension, or hybrid fixation. Regardless of the implant chosen for fixation, secure fixation is paramount to avoid graft displacement and to allow graft integration into the bone tunnel [3,4].

The choice of implant, such as polyether ether ketone (PEEK) interference screws that offer secure fixation with reduced complication risks and MRI compatibility, and bioabsorbable screws, which naturally degrade, minimizes complications [3,5]. In a study by Devandri et al., the ACL reconstruction procedure in Indonesia was limited due to a low budget for reimbursement of the national insurance program. Implants constitute a big amount of the ACL reconstruction budget [6]; hence, a practical and affordable implant may be a solution to this problem. In this study, we aimed to compare the functional outcomes of modified metal fixation with those of biostable (PEEK interference screws) and biodegradable screws.

## 2. Materials and Methods

### 2.1. Study Design

We evaluated the functional outcomes of ACL reconstruction using various fixation techniques in a retrospective cohort analysis. The effectiveness of PEEK interference screws, bioabsorbable interference screws, and modified metal implants in producing favorable postoperative results was evaluated through a comparative analysis. The Institutional Review Board and Ethics Committee approved the study protocol (159/UN4.6.4-5.31/PP36/2024). Written informed consent was obtained from the patient for publication of this research and accompanying images. This study was registered in the Research Registry Database. The study protocol was documented using the STROCSS criteria and complied with the ethical requirements outlined in the 2013 Declaration of Helsinki [7].

### 2.2. Setting and Population

This study was conducted between September 2023 and February 2024. The study population comprised of patients who underwent arthroscopic ACL reconstruction surgery at the aforementioned healthcare facilities.

### 2.3. Inclusion and Exclusion Criteria

The inclusion criteria were patients diagnosed with unilateral ACL rupture between the ages of 17 and 50 years, undergoing their first ACL reconstruction surgery and utilizing a hamstring graft. Patients with preexisting lower extremity complications, history of knee surgery, associated meniscus injury, multiligament knee injuries, congenital knee disorders, pregnant women, knee joint infections, grade III and IV knee osteoarthritis, repeated ACL injuries, or psychiatric disorders were excluded from the study.

### 2.4. Data Collection

The medical records of eligible patients who underwent surgery between February 2023 and September 2023 were accessed to procure demographic information and surgical details, encompassing age, sex, site of injury, date of surgery, and type of implant utilized. Functional outcomes were assessed using the Indonesian Lysholm Knee Scoring Scale and the International Knee Documentation Committee (IKDC) score questionnaire [8,9]. The Lysholm Knee Scale (ranging from 0 to 100) evaluates eight key areas: limp, use of a cane or crutches, locking sensation in the knee, giving way sensation from the knee, pain, swelling, climbing stairs, and squatting swelling [8,10]. The IKDC scoring consists of 10 items evaluated across a range of values and is comprised of three categories: subjective, objective, and additional information regarding the patient’s surgical history [11,12,13]. The data were collected from each patient 6 and 12 months after ACL reconstruction surgery.

### 2.5. Operative Technique

All of the patients underwent single-bundle reconstruction under anesthesia. The hamstring graft was harvested in the same manner for all patients, with a 3 cm longitudinal skin incision over the pes anserine, 1–2 cm medial and distal to the tibial tuberosity. After adequately exposing the insertion site, the hamstring tendon was harvested using a stripper. The superficial fascia and fat of the hamstring were removed, and the rough edges were trimmed appropriately and meticulously. The hamstring was then folded to obtain a 2-strand graft and whip-stitched at each end with a No. 2 polyester suture (Ethibond Excel^®^). The diameter of the graft was measured using a sizing cylinder in 0.5 mm increments. This study did not include grafts with an estimated final diameter of less than 7 mm.

The tibial and femoral tunnels were prepared in the same manner for all patients. The tibial tunnel was standardized to a 50° angle, and the femoral tunnel was drilled through the anteromedial portal (transportal technique) to obtain the most anatomical position of the graft. Finally, the prepared hamstring grafts were implanted and fixed.

**Figure 1:**
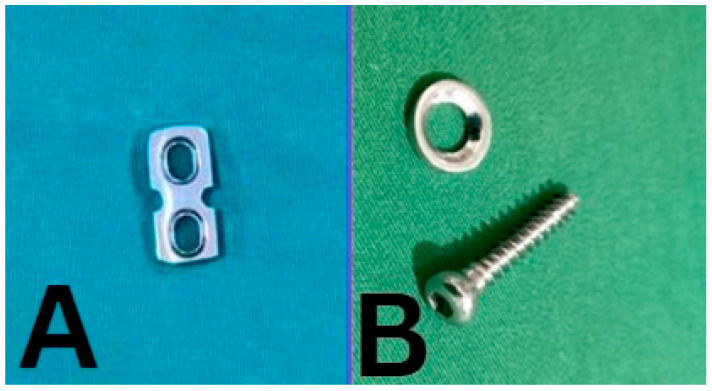
Modified metal implant. (**A**) The modified 2-hole 4.5 mm reconstruction plate and (**B**) 4.5 mm cortical screw and washer.

The patients were divided into three groups. The modified implant group used a modified 2-hole 4.5 mm stainless steel reconstruction plate for femoral fixation and a 4.5 mm stainless steel cortical screw combined with a stainless steel washer for tibial fixations (Figure 1). The PEEK group used a PARCUS GFS mini-suture loop for femoral fixation and a PEEK CF interference screw for tibial fixation. The bioabsorbable group used the pull-up fixation system for the femoral fixation and Endo Ligafix interference screws for tibial fixation. Following surgery, all patients were promptly referred to the physical rehabilitation division and underwent a non-accelerated ACL Reconstruction Rehabilitation protocol.

### 2.6. Data Analysis

Statistical analysis was performed using SPSS software, version 26.0. The Shapiro–Wilk test was utilized for normality assessment and analysis of variance was used for comparative analysis of postoperative outcomes among the fixation groups. A *p* value less than 0.05 is considered statistically significant.

## 3. Results

### 3.1. Sample Characteristics

**Figure 2:**
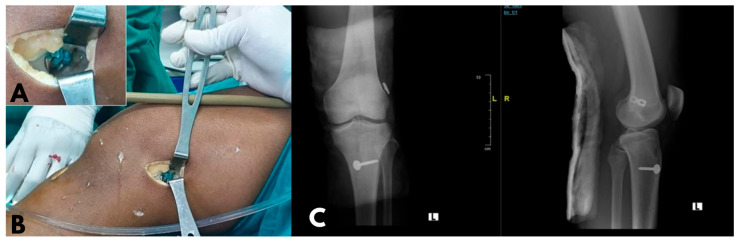
Anterior cruciate ligament reconstruction using a modified implant. (**A**) Modified reconstruction plate being placed on the femoral site. (**B**) A well-fixed modified implant at the femoral site is shown. (**C**) An anterior–posterior–lateral knee radiograph post-operation using the modified implant is shown.

A total of 35 patients underwent arthroscopic ACL. Initially, 10 patients were assigned to the modified implant group (Figure 2), 10 to the PEEK group, and 15 to the bioabsorbable group. In the bioabsorbable group, two patients were lost to follow-up because they were unreachable and did not return for clinical follow up.

Of the 33 patients, the average age at the time of surgery was 26.5 ± 6.5 years (range 18–41). A total of 10 (30.3%), 10 (30.3%), and 13 (39.4%) patients in the modified implant, PEEK, and bioabsorbable groups, respectively, were included in the study. Eight males and two females each were included in the modified implant and PEEK groups, whereas all thirteen patients were male in the bioabsorbable group. Twenty patients underwent reconstructive surgery on their right knee and thirteen on the left knee. The average patient follow-up time was 12.30 ± 0.24 months (range 12.1–13.0). Table 1 summarizes the characteristics of the patients included in this study.

### 3.2. Lysholm Score

At the time of 6-month follow-up, the PEEK group had the highest average Lysholm Score compared to the other groups (85.3 ± 4.42), followed by the modified implant group (80.50 ± 9.50) and the bioabsorbable group (78.85 ± 7.96), with a total average of (81.30 ± 7.89).

At 12-month follow-up, the PEEK group continued to have the highest average Lysholm Score compared to the other groups (89.7 ± 2.75). However, the average Lysholm scores between the groups at 6 and 12 months were not statistically significant (*p* = 0.14 and *p* = 0.77, respectively). The results of the Lysholm score analysis are shown in Table 2.

### 3.3. IKDC Score

Functional outcome comparisons were conducted using the IKDC scores at 6 and 12 months. The PEEK group had the highest average compared to the other groups at 6 months (82.60 ± 5.91) and 12 months (94.00 ± 4.37). However, the difference in average IKDC scores was not statistically significant (*p* = 0.150 and *p* = 0.200 at 6 and 12 months, respectively). The results of the IKDC score analysis are shown in Table 2.

## 4. Discussion

In this study, we conducted an evaluation of 33 patients who underwent ACL reconstruction surgery using three different fixation methods, including bioabsorbable, PEEK, and modified metal implant. The highest Lysholm and IKDC scores, both at 6 months and 12 months following ACL reconstruction, were found in the PEEK group. Nevertheless, there was no significant difference in the functional outcomes between the three groups based on the Lysholm and IKDC scores, both at 6 months (*p* = 0.140 and *p* = 0.150, respectively) and 12 months (*p* = 0.770 and *p* = 0.200, respectively). This finding was consistent with previous studies evaluating different fixation methods for ACL reconstruction. A study conducted by Shumborski et al. examined 133 patients who had arthroscopic ACL reconstruction using four-strand hamstring autografts. These patients were randomly assigned to receive either titanium or PEEK interference screws for femoral and tibial tunnel fixation. The results of this study showed that there were no significant differences in Lysholm and IKDC scores between the two groups. Furthermore, no notable differences were found in the frequencies of graft rerupture, contralateral ACL rupture, and objective outcomes, such as graft incorporation and cyst formation, as evaluated through MRI [14].

In another study, Chao Shen et al. demonstrated comparable outcomes in terms of the IKDC final score (RR, 0.87; *p* = 0.63; 300 patients in five studies) and the Lysholm score (SMD, 0.03; *p* = 0.89; 204 patients in four studies) between bioabsorbable screws and metal interference screws, with bioabsorbable screws associated with higher rates of knee effusion [15]. This study included 10 trials involving a total of 790 patients who underwent single-bundle ACL reconstruction. Furthermore, Xu et al. found no difference in Lysholm and IKDC scores between PEEK and bioabsorbable interference screws. Similarly, there was not any significant contrast in subjective knee joint function and knee laxity between bioabsorbable and metallic screws. However, the analysis highlighted a crucial point: while both options showed comparable functional outcomes initially, metallic interference screws appeared to have fewer reported complications, according to the literature [16]. In addition, Emond et al. concluded that bioabsorbable screws and metal implants yielded statistically similar outcomes based on IKDC, Lysholm, Tegner activity scores, and laxity testing with arthrometry in ACL reconstruction [17].

These collective findings emphasize the need to consider both clinical effectiveness and complication rates when selecting an appropriate fixation method for ACL. Modified metal implants can provide good functional outcomes for graft fixation and offer biomechanical effects similar to those of bioabsorbable and PEEK screws. Metal fixation methods offer reliable stability and strength for a graft, contributing to successful ACL reconstructions. The use of metal interference screws for graft fixation has been associated with improved clinical outcomes and reduced complications compared to other fixation techniques [18]. Furthermore, metal fixation provides a secure attachment of the graft to the bone, promoting proper healing and integration of the graft within the joint [19]. This stability is crucial for the success of ACL reconstruction procedures, ensuring that the graft can withstand the forces and stresses placed on the knee during daily activities and sports participation.

Functional outcome scores improved when the modified implant was used. The average Lysholm score at 6 months was 80.50 ± 9.50, which increased to 87.90 ± 5.50 at 12 months. According to the IKDC scores, the modified metal implant had a functional outcome of 79.90 ± 9.82 (fair) at 6 months, which increased to 90 ± 5.59 (excellent) at 12 months. The findings from this study provide compelling evidence that the utilization of modified metal implants does not impede the healing of grafts. This observation aligns seamlessly with the established theory concerning the phases of ACL graft healing post-reconstruction. According to this theory, patients typically experience a gradual improvement in functional outcomes over time, which correlates with the distinct phases of ACL graft healing. These phases include the following: (1) graft necrosis and hypovascularization, (2) remodeling and vascularization, and (3) ligamentization. Remarkably, this study suggests that after 12 months, the overall ACL healing phase exhibits adaptation processes akin to those observed in the biological ACL [20].

This revelation holds significant implications for ACL reconstruction procedures. By demonstrating the compatibility of modified metal implants with the natural healing process of ACL grafts, the study opens doors to novel possibilities in surgical interventions. It implies that such implants could serve as a viable alternative in ACL reconstruction, offering surgeons and patients a promising avenue for achieving optimal functional outcomes while potentially minimizing complications associated with traditional methods. Thus, this study underscores the importance of continued exploration and innovation in orthopedic surgery to enhance patient care and treatment efficacy.

The utilization of modified metal implants in ACL reconstruction not only demonstrates efficacy but also presents a cost-effective solution, particularly significant in regions with constrained healthcare budgets like Indonesia. The Indonesian Ministry of Health has standardized tariffs for ACL reconstruction procedures, ranging from IDR 9,586,400 to IDR 31,379,700 (equivalent to USD 677 to USD 2215, based on 2019 exchange rates), depending on factors such as region, room class, and hospital type. Importantly, these costs do not encompass accompanying injuries often associated with ACL reconstructions. [6]. In contrast, costs in other countries such as the United States (USD 7129), Sweden (USD 5760), Switzerland (USD 7391), Malaysia (USD 4354), and Thailand (USD 5710) are notably higher. In Indonesian hospitals, where average implant usage is around USD 1387.8, opting for modified metal implants emerges as a pragmatic approach to alleviate financial burdens on patients while ensuring quality treatment [6,21,22,23].

Moreover, the implementation of modified metal implants streamlines treatment pathways, which is particularly beneficial in regions with logistical challenges. In areas where transportation and geographical barriers hinder medical service delivery, the affordability and accessibility of modified metal implants overcome obstacles associated with complex surgical interventions. This makes them well-suited for healthcare facilities with limited budgets, optimizing fund allocation while ensuring timely and effective treatment for ACL injuries.

In summary, the adoption of modified metal implants not only offers cost-effective solutions but also addresses unique challenges faced by healthcare systems in remote and resource-limited regions. By leveraging these implants, healthcare providers can enhance the accessibility and affordability of orthopedic care, ultimately improving patient outcomes and quality of life in underserved communities.

Our study had several limitations. This study had a small sample size, which may have affected the results. In addition, this study used a retrospective design, which has a lower degree of evidence than that of prospective studies and randomized clinical trials. Future research should focus on expanding the sample size, targeting specific patient populations (e.g., athletes), and extending the follow-up duration to provide further insights into the comparative efficacy of fixation methods in ACL reconstruction. Additionally, exploring the biomechanical properties and long-term durability of modified metal implants warrants further investigation to optimize treatment outcomes in patients undergoing ACL reconstruction.

## 5. Conclusions

This study found that modified metal implants, along with PEEK and bioabsorbable implants, are viable options for ACL reconstruction. The choice of fixation method should be tailored to individual patient characteristics and preferences, considering biomechanical stability and long-term outcomes.

## Figures and Tables

**Table 1:** Patient characteristics.

Indicator		N	Mean ± SD
Age (year)			26.50 ± 6.50
Gender	Male	29 (87.9%)	
	Female	4 (12.1%)	
Implant	Modified implant group	10 (30.3%)	
	PEEK group	10 (30.3%)	
	Bioabsorbable group	13 (39.4%)	
Affected Knee (Right/Left)	Modified implant group	5/5	
	PEEK group	6/4	
	Bioabsorable group	9/4	
Follow up time (month)			12.30 ± 0.24

SD, standard deviation; PEEK, polyether ether ketone.

**Table 2:** Comparison of the three types of implants based on the Lysholm and IKDC scores.

	Lysholm Score (Mean ± SD)		IKDC Score (Mean ± SD)	
	6 months	12 months	6 months	12 months
Modified implant group	80.50 ± 9.50	87.90 ± 5.50	79.90 ± 9.82	90.00 ± 5.60
PEEK group	85.3 ± 4.42	89.70 ± 2.75	82.60 ± 5.91	94.00 ± 4.37
Bioabsorbable group	78.85 ± 7.96	89.07 ± 2.75	76.46 ± 6.04	90.85 ± 5.41
*p*-value	0.140	0.770	0.150	0.200

SD, standard deviation; IKDC, International Knee Documentation Committee; PEEK, polyether ether ketone.

## Data Availability

Dataset available upon request from the authors.
